# A Novel Mutation in *ERCC8* Gene Causing Cockayne Syndrome

**DOI:** 10.3389/fped.2017.00169

**Published:** 2017-08-09

**Authors:** Maryam Taghdiri, Hassan Dastsooz, Majid Fardaei, Sanaz Mohammadi, Mohammad Ali Farazi Fard, Mohammad Ali Faghihi

**Affiliations:** ^1^Genetic Counseling Center, Shiraz Welfare Organization, Shiraz, Iran; ^2^Comprehensive Medical Genetic Center, Shiraz University of Medical Sciences, Shiraz, Iran; ^3^Department of Medical Genetics, Shiraz University of Medical Sciences, Shiraz, Iran; ^4^Center for Therapeutic Innovation, Department of Psychiatry and Behavioral Sciences, Miller School of Medicine, University of Miami, Miami, FL, United States

**Keywords:** *ERCC8*, Cockayne syndrome, novel mutation, rare inherited disorders, neurodevelopmental disorders, intellectual disability

## Abstract

Cockayne syndrome (CS) is a rare autosomal recessive multisystem disorder characterized by impaired neurological and sensory functions, cachectic dwarfism, microcephaly, and photosensitivity. This syndrome shows a variable age of onset and rate of progression, and its phenotypic spectrum include a wide range of severity. Due to the progressive nature of this disorder, diagnosis can be more important when additional signs and symptoms appear gradually and become steadily worse over time. Therefore, mutation analysis of genes involved in CS pathogenesis can be helpful to confirm the suspected clinical diagnosis. Here, we report a novel mutation in *ERCC8* gene in a 16-year-old boy who suffers from poor weight gain, short stature, microcephaly, intellectual disability, and photosensitivity. The patient was born to consanguineous family with no previous documented disease in his parents. To identify disease-causing mutation in the patient, whole exome sequencing utilizing next-generation sequencing on an Illumina HiSeq 2000 platform was performed. Results revealed a novel homozygote mutation in *ERCC8* gene (NM_000082: exon 11, c.1122G>C) in our patient. Another gene (*ERCC6*), which is also involved in CS did not have any disease-causing mutations in the proband. The new identified mutation was then confirmed by Sanger sequencing in the proband, his parents, and extended family members, confirming co-segregation with the disease. In addition, different bioinformatics programs which included MutationTaster, I-Mutant v2.0, NNSplice, Combined Annotation Dependent Depletion, The PhastCons, Genomic Evolutationary Rate Profiling conservation score, and T-Coffee Multiple Sequence Alignment predicted the pathogenicity of the mutation. Our study identified a rare novel mutation in *ERCC8* gene and help to provide accurate genetic counseling and prenatal diagnosis to minimize new affected individuals in this family.

## Introduction

Cockayne syndrome (CS), which was first reported by Edward Alfred Cockayne, a British physician, is a rare early-onset, progressive neurological disorder characterized by generalized growth retardation, which manifests as cachectic dwarfism, microcephaly, extreme cutaneous photosensitivity, partial hearing loss, facio-skeletal and/or gait abnormalities, and retinopathy. Affected individuals often have disproportional long extremities, joint contracture, intellectual disability, and progressive neurodegeneration leading to death, usually around 8 years. Regarding the neurological findings, the brain in the affected individuals shows neuronal dystrophy, an increase in fibrosis, and an accumulation of senile plaques and/or neurofibrillary tangles along with progressive demyelination or dysmyelination. A number of studies have been conducted concerning this disorder, and they have reported that the disease is caused by homozygous or compound heterozygous mutations in either *ERCC Excision Repair 6* (*ERCC6*) gene (also known as *CSB*) or the *ERCC8* gene (also known as *CSA*) (1–5). This syndrome shows a different age of onset and rate of progression with broad range of severity from severe form with manifestation of abnormalities at birth or in the early neonatal period to mild and late-onset presentations. Due to the progressive nature of this disease, its diagnosis should be considered at any stage of the disease (6, 7). Therefore, molecular genetic analysis of the genes involved in this disorder can be very useful to confirm the suspected cases of CS. Here, we report a novel mutation in *ERCC8* gene in a 16-year-old boy affected by characteristic abnormalities of CS.

## Case Presentation

**Figure 1:**
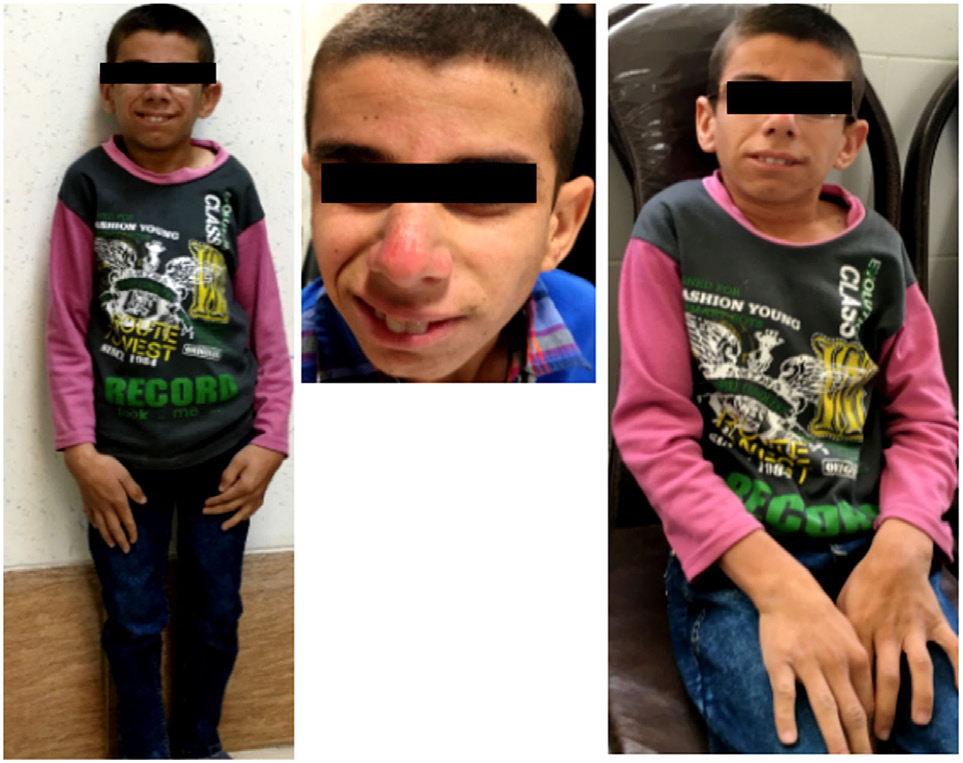
Clinical presentation; proband is 16 years old boy with poor general growth, short stature postnatal microcephaly, lean cachectic face, and disproportionately large hands and ears. Inset images show large hand and evidences of cutaneous photosensitivity.

A 16-year-old boy was referred to Genetic Counseling Center in Shiraz Welfare organization (Shiraz, Iran) with chief complaint of general growth and developmental delay, including poor weight gain and short stature (Figure 1). He was born to a consanguineous first-degree relative family by normal vaginal delivery with normal APGAR score at 1 min (score 9), 5 min (score 10), and 10 min (score 10); normal weight (*Z* score: −1.7); and normal head circumference (*Z* score: −1.1). The parents noted the onset of cutaneous photosensitivity at the age of nine months and intellectual disability and poor weight gain after 2 years of his life. The family noted mild to moderate intellectual disabilities, delay in mental milestones, learning difficulties, finding directions, and poor memory. Currently, at the age of 16 years, in physical exam, he looks thin (20 kg, *Z* score: −10.47), short in stature (height of 118 cm, *Z* score: −6.1), and he has small chin, aged face, and sunken eyes. In addition, he has microcephaly (*Z* score: −6.7), and he often has skin redness over the nose or other exposed body area following exposure to sun. Moreover, he has disproportionately large ear, large hand, fine tremor, as well as upper and lower extremity joint contracture with normal muscle power, and deep tendon reflexes are mildly increased. Paraclinical tests were also performed, which revealed a normal male C-banded karyotype and a negative fragile X syndrome triple repeat expansion test. In addition, brain MRI at the age of 8 years showed demyelination and diffuse white matter changes in both cerebral hemispheres suggestive of leukodystrophy, dilated ventricular system, and slight cortical atrophy.

**Figure 2:**
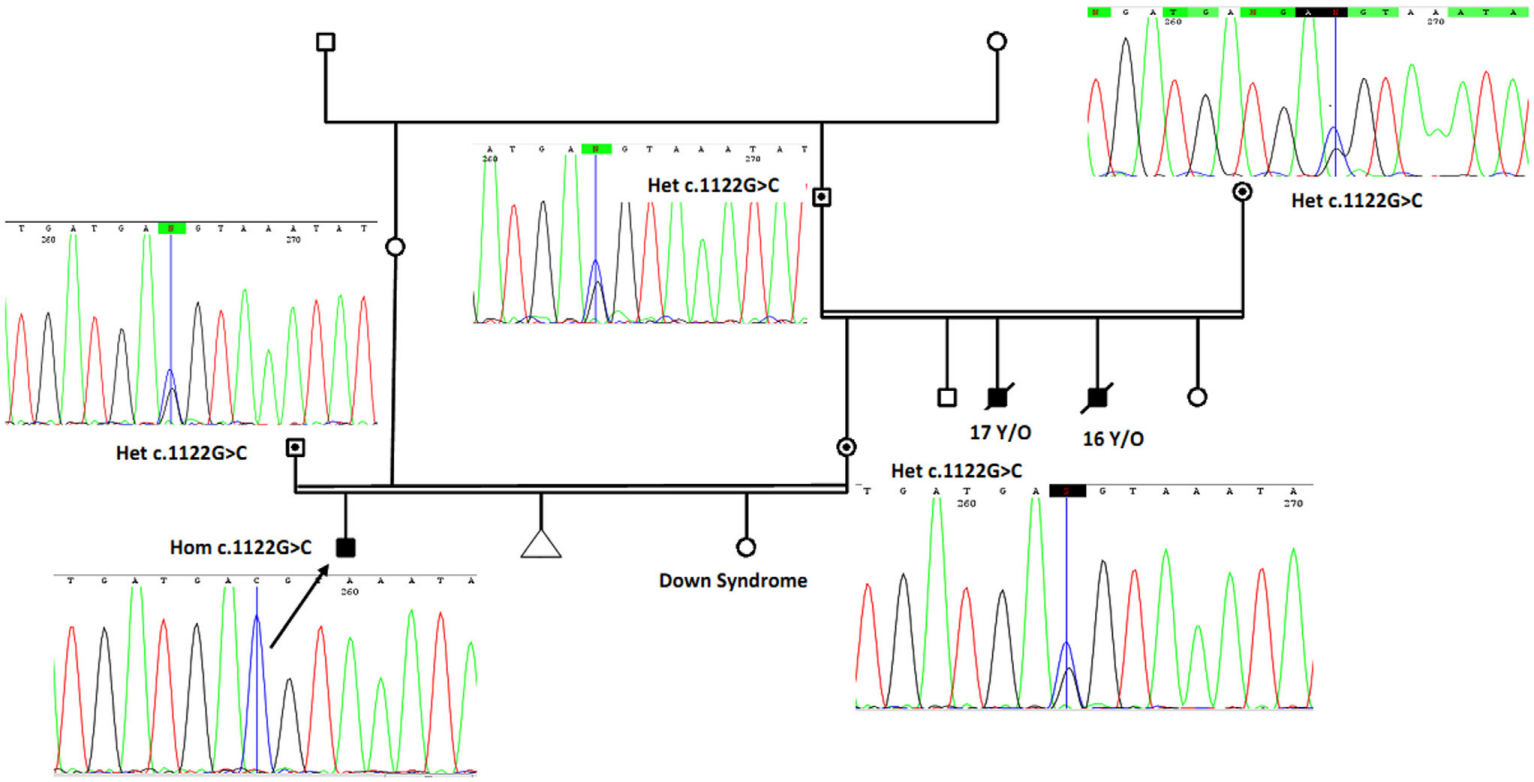
The proband (marked by arrow and confirmed using Sanger sequencing as homozygote for novel mutation) is a boy with cutaneous photosensitivity, severe growth retardation and intellectual disability. Parents have consanguineous marriage, and two of his uncles from mother side had similar phenotype and died around 16 and 17 years. The proband sister is a known case of Down syndrome. By using Sanger sequencing, parents and grandparents from mother side were confirmed as heterozygous carriers of the identified *ERCC8* gene mutation and are marked with solid circles with corresponding sequencing chromatograms. Het, heterozygote; Hom, homozygote.

Two uncles from mother side had similar but more severe symptoms and died below the age of 17 years. In addition, there is one case of documented trisomy 21 (Down syndrome) in the family (Figure 2). Carriers in his family and relatives have normal developmental features without photosensitivity. Family members of the patient gave informed consent before undergoing DNA test based on the requirements of the ethics committee in Comprehensive Medical Genetics Center, Shiraz University of Medical Sciences.

## Next-Generation Sequencing

Whole exome sequencing was utilized for capture, enrichment, and sequencing of all exons of protein-coding genes as well as some important other genomic regions, utilizing an Agillent V.5 whole exome capture kit. DNA sample from proband was sequenced, using Illumina HiSeq2000 machine, by standard Illumina protocol for pair-end 99-nucleotide sequencing. Next-generation sequencing was performed to sequence close to 100 million reads on Illumina HiSeq2000 Sequencer. In general, test platform examined >95% of the targeted regions with sensitivity of above 99%. In this test, point mutations and microinsertion/microdeletions and duplication (<20 bp) can be simultaneously detected. Bioinformatics analysis of the sequencing results were performed using BWA aligner (8), GATK (9), and ANNOVAR (10) open access software as well as public databases and standard bioinformatics software. The text files of sequences were aligned using BWA aligner tool, and variants were identified using GATK and annotated with the use of ANNOVAR software. In total, >120 K annotated variants were identified with hetero/homo ratio of 1.6:1.8, which then were filtered based on their frequency, location, functional consequences, inheritance pattern, and more importantly clinical phenotype. Result revealed a rare novel homozygous mutation in *ERCC8* gene (NM_000082: exon 11: c.1122G>C) in the proband, but there was not identified any mutations in *ERCC6* gene.

## Sanger Sequencing and Segregation Studies

To confirm the novel mutation, peripheral blood samples were obtained from the family member of the proband and other extended family members. Genomic DNA was then extracted from these samples using QIAamp DNA Mini Kit (Qiagen). Oilgonucleotide primers were used to amplify exon 11 of *ERCC8* gene as well as flanking intronic sequences. Amplified DNA was then sequenced with both forward and reverse primers using Sanger sequencing reagent from Applied Biosystems™. Sanger sequencing data were analyzed using 4Peaks free software, which confirmed homozygote mutation in the proband and heterozygote in his parents and grandparents (Figure 2). To predict pathogenicity of the novel mutation, we used different bioinformatics software and approaches and following evidences are supportive of the disease-causing feature of the mutation.

**Figure 3:**
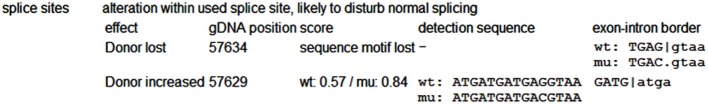
Prediction of splice site changes using MutationTaster with the use of NNSplice database. As can be seen in this figure, due to the mutation in the position of 57634 in gDNA sequence, the lost of donor sequence motif has occurred and an increase for a donor site in position of 57629 has been predicted, and due to these changes, normal splicing may be disturbed. MutationTaster determines the position of splice site change relative to intron/exon borders: if a loss/decrease of a splice site occurs at an intron/exon border or exon/intron border, this will be taken for a “real” splice site change. A gain of a completely new splice site is displayed, if the confidence score of the newly created splice site is > 0.3. An increase in an already existing splice site will be displayed if the change in the confidence score is >10%. All changes are displayed with the effect, genomic position of the splice site, the prediction score for wild-type (wt) and/or mutated (mu) splice site as generated by NNSplice, the (wt) detection sequence itself and very short sequence information about the splice site, with a pipe (|) indicating the border between intron and exon.

**Figure 4:**
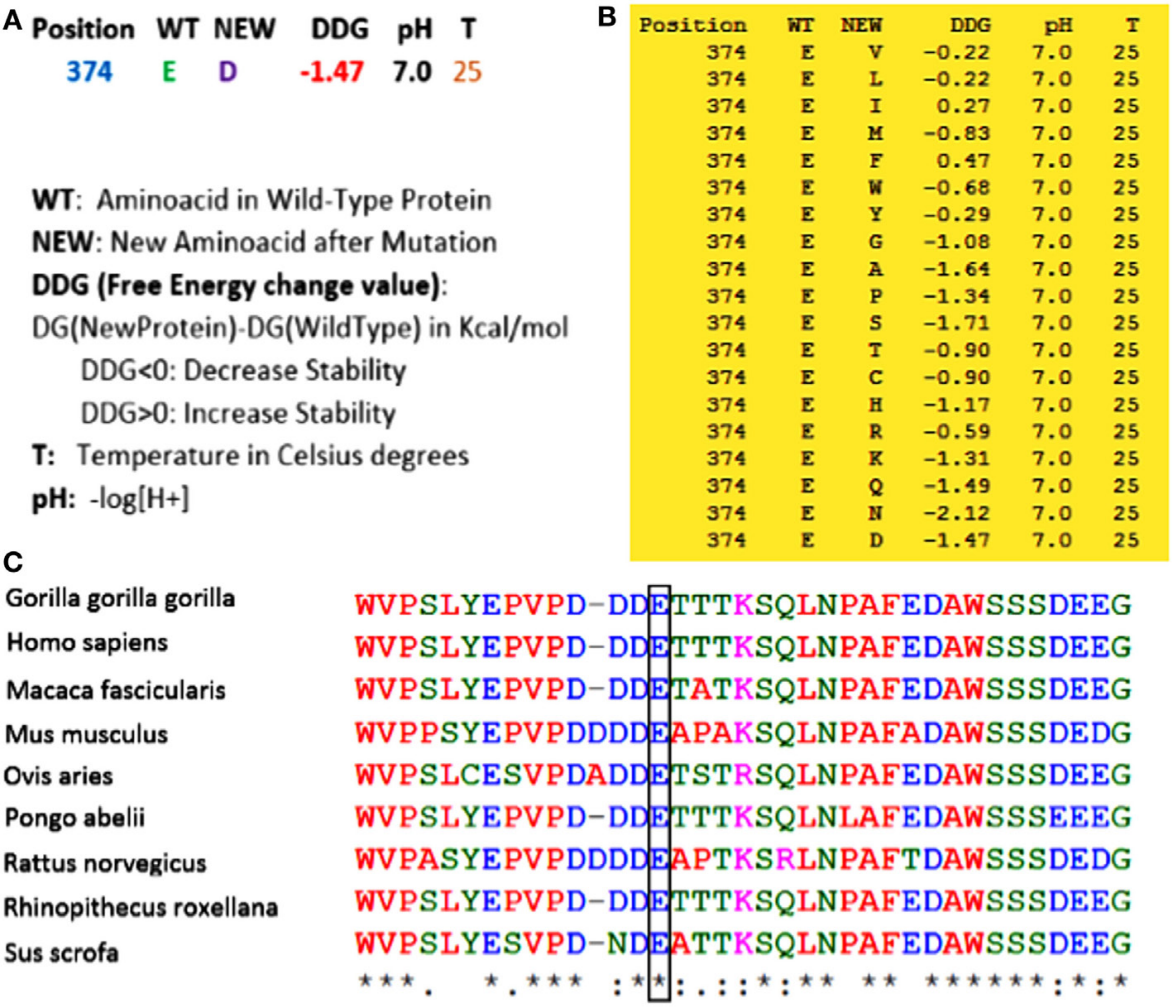
**(A)** By using I-Mutant v2.0, it was revealed that with the substitution from Glu 374 to Asp, the stability of the protein will be decreased. **(B)** All alterations of stability for all other amino acids in this residue were also predicted by this program. As can be seen, most of the substitutions in this region can decrease the stability of the protein. **(C)** Comparative amino acids alignment of ERCC8 protein across Kingdoms shows that the Glu374 residue is highly conserved during evolution. The conserved Glu (E) residue is shown in the rectangular box. Protein sequences were obtained from National Center for Biotechnology. Symbols: (*)—identical amino acids; (:)—just similar amino acids.

(1) Whole exome sequencing only identified this homozygous mutation in *ERCC8* gene (not in another gene involved in this syndrome) to be the main cause of CS in the proband. (2) As shown in Figure 2, using Sanger sequencing, the mutation was confirmed in the proband, and based on the heterozygote mutation in his parents and grandparents, the inheritance pattern must be an autosomal recessive mode. (3) MutationTaster, an important online software designed for prediction of damaging effects of alternative amino acids, predicted that this variation will be damaging. (4) Distance to splice site is one nucleotide, and on the basis of information obtained from MutationTaster program, splice site may be changed due to this nucleotide change (Figure 3), and alteration within used splice site is likely to disturb normal splicing (Figure 3). MutationTaster uses a locally installed third-party splice site prediction program, namely *NNSplice* from the Berkeley Drosophila Genome Project (a web-based version is available at http://fruitfly.org/seq_tools/splice.html) to analyze possible changes in splice sites. (5) Using I-Mutant v2.0 (Predictor of Protein Stability Changes upon Mutations), it was revealed that protein stability will be decreased upon the change of Glu 374 to Asp (Figure 4A). Other substitutions in this residue were also predicted in term of protein stability (Figure 4B). (6) As can be seen in Figure 4C, the comparative amino acids alignment of ERCC8 protein across Kingdoms using T-Coffee Multiple Sequence Alignment Program revealed that this amino acid is highly conserved during evolution. (7) Genomic Evolutionary Rate Profiling (GERP) conservation score predicted a score 4.350 for this residue and as determined by this program that its score ranges from −12.3 to 6.17, with 6.17 being the most conserved, can confirm the conservation of this amino acid. (8) Combined Annotation Dependent Depletion (CADD) showed a score of 11.740 for this change, which predicted its deleterious effects. CADD is a tool for scoring the damaging effects of single nucleotide variants as well as insertion/deletions variants in the human genome. The CADD score combines information from 63 different annotations including PhastCons, GERP, PhyloP, SIFT, and PolyPhen, using a support vector machine classifier. It measures deleteriousness by using observed variant frequency as the basis for its calculation. The score ranges from 1 to 99, with a higher score indicating greater deleteriousness. Values ≥10 are predicted to be the 10% most deleterious substitutions, ≥20 indicate the 1% most deleterious. (9) The PhastCons, which is a program for identifying evolutionarily conserved elements in a multiple alignment, identified a score 1 for this residue (a number between 0 and 1 describes the degree of sequence conservation among 17 vertebrate species) and confirmed the high conservation of this amino acid. On the basis of these data, c.1122G>C variant in the last nucleotide of exon 11 of *ERCC8*, which is highly conserved in the 5′ splice site, is likely to be pathogenic in our patient affected by CS. Since we did not have access to the samples for qRT-PCR to prove splicing defect causing by the identified mutation, we performed NGS sequencing again on patient sample and reanalyzed the new set of data to find out if any other mutation can be identified to explain the clinical phenotype. Even on our new set of sequencing data, we only identified ERCC8 mutation (c.1122G>C), as causative, which has complete phenotypic overlap with the observed phenotype. Although, we would not be able to experimentally comment on splicing defect, pathogenicity of the identified mutation is clear based on segregation studies and two times NGS sequencing. Therefore, the variant is likely to be missense mutation, but splicing defect cannot be entirely ruled out.

## Discussion

Cockayne syndrome is a developmental multisystem disorder, with non-homogeneous clinical phenotype (6, 11–13) and is considered as a progeria, and many of the clinical features, including early-onset neurodegeneration, and skin appearance resemble accelerated aging (14). In a comprehensive study conducted by Wilson et al. (15), it was proposed considering three main factors to diagnose CS, which include growth failure, microcephaly, and developmental delay. They suggested that CS should be suspected in any child with postnatal growth failure, microcephaly, and two of the following features: persistently cold hands and feet, bilateral deafness, an increased sensitivity to sunlight (intention) tremor, joint contractures, and progressive loss of body fat, cataracts, or characteristic facial features. The mean age at death was reported to be 8.4 years, which is more frequently due to progressive neurodegeneration. Overlap with xeroderma pigmentosum (XP) has been reported since patients usually have cutaneous photosensitivity or other more severe skin disorders (16). Therefore, molecular genetic testing can be very useful to confirm the genetic cause of the disease.

Mutations in *ERCC* genes have been linked to CS (17). There are more than 39 documented pathogenic ERCC8 mutations registered so far at the Human Gene Mutation Database (HGMD), including 11 missense/nonsense mutations, 7 splicing variants, 1 small deletion, and 2 insertions. In addition, 90 pathogenic mutations in *ERCC6* gene have been reported in HGMD, mainly missense/nonsense (around 35 variants). In our study, we noticed several symptoms due to accelerated aging in general and progeria in facial appearance of the proband, but heterozygous carriers of the disease are apparently normal and has no significant medical problem to report.

In CS, defective nucleotide excision repair (NER) of oxidized genomic DNA leads to accelerated aging (12). The NER pathway, which is a highly evolutionarily conserved repair mechanism, is one of the predominant, and perhaps universal, mechanism that contributes to preservation of genomic integrity. At least 20–30 proteins have been proposed to have key roles in the pathway in a sequential pattern. The basic process involved in this pathway, to repair DNA damages as the distinct helical distortion caused by UV-induced photoproducts, consists of four steps: first, site-specific DNA lesion recognition and demarcation, which requires different factors, mainly three proteins—the XP complementing proteins DDB1 (XPE) and XPC and RD23B–centrin 2; second, the removal of the damaged site of DNA and certain adjacent sequences by the DNA excision repair proteins ERCC5 (XPG; 3′-endonuclease) and ERCC4 (XPF; 5′-endonuclease complexed with excision repair cross-complementing protein 1), leading to the dual incision on either side of the lesion and excision of around 29 nucleotides; third, re-synthesis of the removed damaged site using the second strand as a template; in the final step, the newly synthesized portion is ligated to the existing downstream sequence to complete the repair process, creating the repaired DNA strand (18–20). However, there is another NER pathway that specifically operates on the transcribed strand of transcriptionally active genes, so-called transcription-coupled NER (TC-NER). Until now, the mechanism of preferential recognition and correction of lesion in a gene that is being transcribed has not been fully understood, while it is proposed that damage recognition factor in NER, XPC, is essential for this alternative NER pathway. In addition, it has been shown that the recognition of damage and repair initiation can be handled by other specific factors, which block RNA polymerase II elongation during gene transcription and dissociating it from the DNA strand allowing repair to proceed (20–26). It is worth noting that there is an important role for the Cockayne’s syndrome genes, *ERCC8* (*CSA*) and *ERCC6* (*CSB*), in this activity (1).

The *ERCC8* gene located on chromosome 5q12.1 encodes a WD repeat protein named Cockayne syndrome A (CSA), which is involved in repairing damaged DNA (27). DNA can be damaged by UV radiation from the sun and by toxic chemicals and free radicals. This damage resulted from these agents can prevent the most important cell functions such as gene transcription. If the damage is not corrected, DNA damage accumulates leading to abnormal functions of cells and can result in the cell death (12, 28, 29). While DNA damage has usually occurred, cells have an ability to repair it before it can cause any deleterious effects. Cells have evolved multiple DNA repair pathways to correct DNA damage; one such pathway involves the CSA protein. This protein specializes to repair damaged DNA of active genes (1). However, its precise role in this process remains poorly understood. Substrate-recognition component of the CSA complex, a DCX (DDB1-CUL4-X-box) E3 ubiquitin–protein ligase complex, plays a key role in TC-NER. In this pathway, CSA interacts with Cockayne syndrome type B protein and with p44 protein, a subunit of the RNA polymerase II transcription factor IIH. The CSA complex (DCX (ERCC8) complex) helps to promote the ubiquitination and subsequent proteasomal degradation of ERCC6 in a UV-dependent manner, and this ERCC6 degradation is essential for the recovery of RNA synthesis after transcription-coupled repair. CSA is required for the recruitment of XAB2, HMGN1, and TCEA1/TFIIS to a transcription-coupled repair complex, which removes RNA polymerase II-blocking lesions from the transcribed strand of active genes. In addition, the CSA protein interacts with other proteins, probably to identify areas of damaged DNA (27, 30–35). It has been identified that cells in CS due to ERCC gene mutations are abnormally sensitive to ultraviolet radiation and are defective in the repair of transcriptionally active genes, leading to deleterious abnormalities in affected individuals by CS (1, 12). Therefore, establishment of an unambiguous diagnosis of CS is essential to manage the patient properly, to help families in caring for affected individuals, and to provide accurate genetic counseling and prenatal diagnosis to minimize new affected persons.

With advances in sequencing technologies, the disease is more readily detectable, and secondary prevention for families affected by this disorder becomes more accessible. While heterozygous carriers are not apparently sick, they are at risk of passing this deleterious mutation into their offspring. Next-generation sequencing panels including *ERCC* gene family members must be requested for kids with childhood cachectic dwarfism, failure to thrive, microcephaly, and cutaneous photosensitivity. The molecular test should also be offered to family members of known patients who intend to have consanguineous marriage. Since there is a high rate of genetic diseases in Iran, mainly in rural areas where first-degree marriages are more common, identifying and reporting the rare novel pathogenic mutations would be extremely useful for secondary prevention of inherited disorders with homozygous pattern of inheritance. With the limited available therapeutic option for most of the CS, molecular diagnosis might enable geneticists and pediatricians to provide informative genetic counseling, perform prenatal diagnosis, and implement prevention measures for such patients. Therefore, genetic counseling should be recommended to all individuals with CS and families for their next pregnancies and for other family members who want to have consanguineous marriages. In our study, we could identify a novel mutation that was offered to the family members of the proband to confirm the segregation of the variant, and our evidences can suggest that this variant can be the genetic cause of the disease in this family.

## Concluding Remarks

In summary, a private novel mutation in *ERCC8* gene was identified in patient affected by CS in southwest of Iran. The mutation has not been previously reported, and this report with different evidences will serve to document its pathogenicity. In addition, we have validated this mutation in parents and other extended family members and concluded that disease phenotype segregated with homozygous genotype.

## Ethics Approval and Consent to Participate

Ethic committee at Shiraz University of Medical Sciences, Comprehensive Medical Genetic center has approved the study and parents of affected individual have signed written consent indicating their voluntary contribution to the current study.

## Author Contributions

MAF conceived and designed the study, assembled and interpreted data, and wrote the manuscript. MT clinically evaluated the patient, interpreted data, and wrote the manuscript. HD designed primers, interpreted data, performed RT-PCR, and wrote the manuscript. MF interpreted data. SM performed the experiments. MAFF performed RNA extraction and cDNA synthesis.

## Conflict of Interest Statement

The authors declare that the research was conducted in the absence of any commercial or financial relationships that could be construed as a potential conflict of interest.
